# Urethral-sparing laparoscopic simple prostatectomy for the treatment of benign prostatic hyperplasia with asymptomatic urethral stricture after urethral stricture surgery

**DOI:** 10.1186/s12894-024-01487-8

**Published:** 2024-04-30

**Authors:** Changhao Hou, Zhiqiang Luo, Nailong Cao, Xiaoyong Hu, Lujie Song, Qiang Fu, Jiong Zhang, Jianwen Huang

**Affiliations:** 1 Department of Urology, Shanghai Sixth People’s Hospital Affiliated to Shanghai Jiao Tong University School of Medicine, Shanghai, 200233 China; 2 Shanghai Eastern Institute of Urologic Reconstruction, Shanghai, 200233 China

**Keywords:** Begin prostatic hyperplasia, Urethral stricture, Laparoscopy Prostatectomy

## Abstract

**Objective:**

To evaluate the efficacy of urethral-sparing laparoscopic simple prostatectomy (US-LSP) for the treatment of large-volume (>80 ml) benign prostatic hyperplasia (BPH) with asymptomatic urethral stricture (urethral lumen > 16 Fr) after urethral stricture surgery.

**Methods:**

We retrospectively analyzed clinical data of 39 large-volume BPH patients with asymptomatic urethral stricture after urethral stricture surgery who underwent US-LSP from January 2016 to October 2021. Postoperative follow-ups were scheduled at 1, 3, and 6 months.

**Results:**

All patients affected by significant BPH-related lower urinary tract symptoms (LUTS) including 22 cases with asymptomatic anterior urethral stricture and 17 cases with asymptomatic posterior urethral stricture. Median operative time was 118 min (interquartile range [IQR]100–145). Median estimated blood loss was 224 ml (IQR: 190–255). 33 patients(84.6%) avoided continuous bladder irrigation. Postoperative complications occurred in 5 patients (12.8%), including 4 cases with Clavien-Dindo grade 1 and grade 2 and 1 case with grade 3a. During follow-up, US-LSP presented statistically significant improvements in LUTS compared to baseline (*P* < 0.05). A total of 25 patients had normal ejaculation preoperatively and 3 patients (12%) complained retrograde ejaculation postoperatively. Two patients (5.1%) reported stress urinary incontinence (SUI) and no patient reported aggravated urethral stricture during follow-up.

**Conclusions:**

US-LSP was safe and effective in treating large-volume BPH with asymptomatic urethral stricture after urethral stricture surgery. Meanwhile, US-LSP could reduce the risk of SUI in patients with asymptomatic posterior urethral stricture and maintain ejaculatory function in a high percentage of patients.

## Introduction

For BPH patients with a history of urethral stricture surgery, once the lower urinary tract symptoms (LUTS) develop, it is crucial to evaluate the patient for recurrent symptomatic urethral stricture or bladder neurogenic pathology from the initial urethral stricture surgery [1]. According to European Association of Urology (EAU) guidelines on urethral strictures, Urethral stricture with a lumen of 16Fr or greater was considered low grade stricture and asymptomatic incidental stricture. Meanwhile, Patients with asymptomatic strictures have a low risk of progression to a high grade stricture and the development of symptoms and none require surgical intervention [2]. Thus, once the recurrent symptomatic urethral stricture and bladder neurogenic etiology have been ruled out, a diagnosis of BPH can be confirmed. BPH with significant LUTS should be treated, while concomitant asymptomatic urethra stricture does not need repeat surgical treatment. Although the first choice of treatment for BPH is pharmacological treatment, 30% of patients still require surgical treatment [3]. Currently, endoscopic techniques represent the first choice for surgery treatment of BPH in the urological armamentarium. For example, transurethral holmium laser enucleation of prostate (HoLEP), as the first choice for large-volume (> 80 ml) BPH, has been widely used in clinical practice [4, 5]. However, for patients with asymptomatic urethral stricture after urethral surgery, it is difficult for the 24Fr or 26Fr resectoscope sheath to enter the prostatic urethra, and rough insertion of the sheath may cause avulsion or damage of the urethral mucosa and aggravate urethral stricture during the endoscopic surgery.

For large-volume BPH, open simple prostatectomy (OP) is still a standard treatment [6]. However, a high risk of perioperative complications associated with OP represents a major limitation [7]. With the development of laparoscopic technology, laparoscopic simple prostatectomy(LSP)as a minimally invasive treatment method has been unanimously recognized for its safety and effectiveness, particularly for large-volume BPH cases or wherever the endoscopic treatment is not available [8, 9].

In 2011, Quan et al. reported that urethral-sparing laparoscopic simple prostatectomy (US-LSP) with preservation of prostate urethra and the bladder neck was a safe and feasible approach for large-volume BPH. Because of the preservation of the prostate urethra and bladder neck, US-LSP had the advantages of lower incidence of urinary incontinence and retrograde ejaculation [10]. In 2018, Wang et al. described, for the first time, urethral-sparing robotic-assisted simple prostatectomy (RASP) via an extraperitoneal approach for BPH treatment [11]. Compared to LSP, RASP has the advantage of a stereoscopic three-dimensional vision and exceptional dexterity to facilitate the more technically demanding surgical steps, while the advantage of minimally invasive surgery is maintained [11, 12]. However, in terms of costs, RASP is more expensive than LSP, because it requires more material costs and a more expensive robotic platform. Therefore, US-LSP was a relatively cheaper technique and particularly suited for BPH patients with asymptomatic urethral stricture for surgeons with vast experience in pelvic laparoscopic surgery. In previous studies, LSP was performed in BPH patients with normal urethra [8–12]. For these patients, endoscopic surgery may also be an option. However, for the BPH patient with asymptomatic urethral stricture, LSP is necessary, and endoscopic surgery may not be feasible. At present, there is a lack of studies on LSP for the treatment of BPH with asymptomatic urethral stricture after urethral stricture surgery. Therefore, our study aims to evaluate, for the first time, the efficacy of US-LSP for treating large-volume BPH with asymptomatic urethral stricture after urethral stricture surgery.

## Patients and methods

### General information

**Fig. 1:**
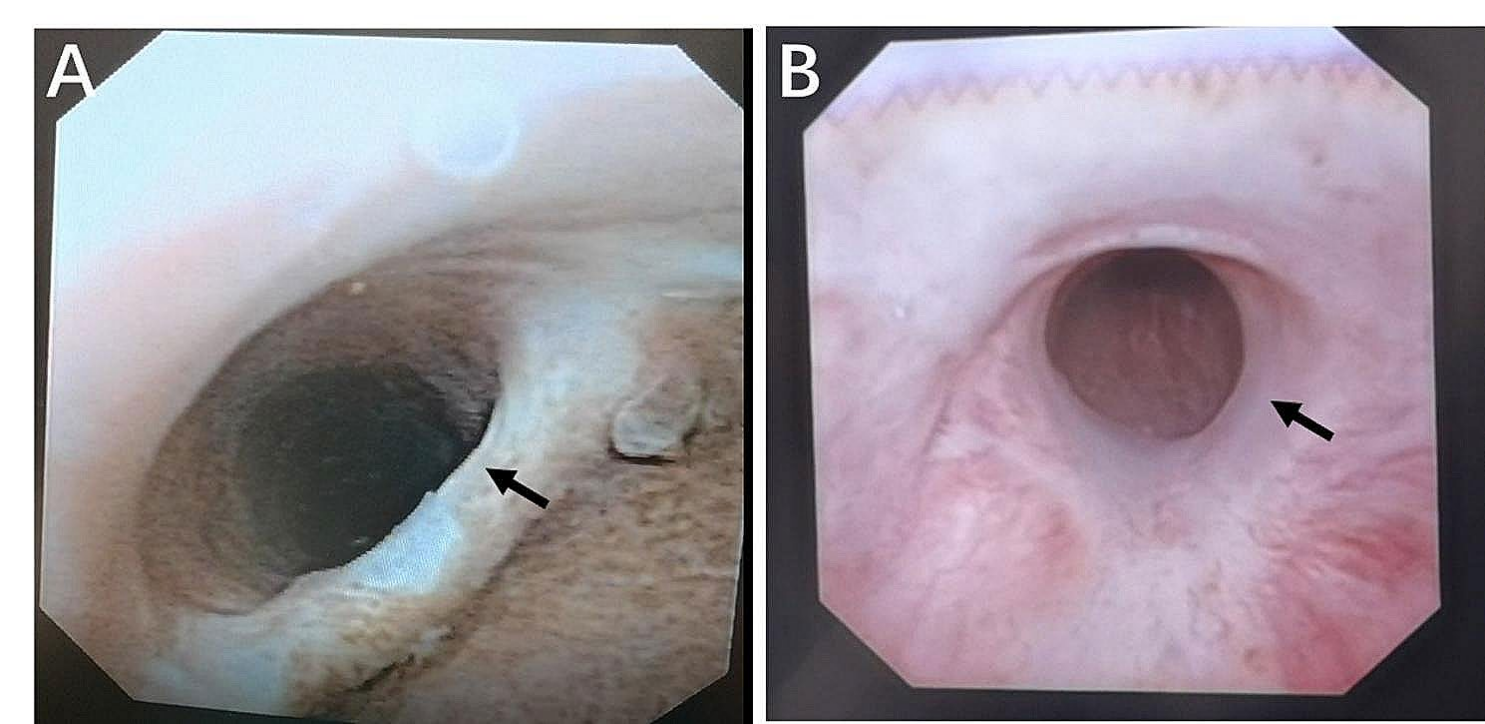
Flexible cystoscopy. Flexible cystoscopy revealed asymptomatic anterior urethral stricture (**A**) and asymptomatic posterior urethral stricture (**B**), respectively (16Fr flexible cystoscopy pass freely through the stenoses in all patients) (arrow)

We retrospectively analyzed the clinical data of 39 large-volume (>80 ml) BPH patients with asymptomatic urethral stricture who underwent US-LSP from January 2016 to October 2021. Any patient with severe urethral stricture, neuro-vesical dysfunction and/or prostate cancer was excluded from the study. All patients had a history of urethral stricture caused by iatrogenic factors, trauma, congenital abnormalities, and inflammation. After successful urethral stricture surgery, which included anastomotic urethroplasty, substitution urethroplasty, and direct vision internal urethrotomy (DVIU), all patients maintained acceptable voiding parameters (maximum urinary flow rate (Qmax) > 15 ml/s and post-void residual (PVR)<50 ml). However, they later developed progressively severe LUTS (International Prostate Symptom Score (IPSS) ≥ 20 points). All patients underwent 16Fr flexible cystoscopy (Olympus Europe, Hamburg, Germany) (Fig. 1) and urodynamic evaluation to exclude the recurrence of symptomatic urethral stricture and neurogenic bladder dysfunction (for example, projected isovolumetric pressure (PlP) <100 is used as a diagnostic criterion for hypocontractile or acontractile detrusor). In other words, 16Fr flexible cystoscopy passes freely through the strictures, and 24Fr or 26Fr resectoscopes do not pass through the strictures. All patients had failed to respond to medical treatment, including alpha-blockers and finasteride (treatment of at least six months). Before US-LSP, all patients signed a dedicated informed consent and approved the use of their data for research purposes. Preoperative data collected included age, volume of prostate, body mass index (BMI), Q_max_, IPSS, Quality of life (QoL), PVR, prostate specific antigen (PSA), and the International Index of Erectile Function-5 questionnaire (IIEF-5) assessment. Additionally, an abridged version of the 25-item Male Sexual Health Questionnaire (MSHQ-EjD Short Form) was used to assess ejaculatory dysfunction (EjD). In the case of elevated PSA, the patients underwent prostate multiparametric magnetic resonance imaging (mp-MRI) to exclude the presence of prostate cancer.

### Surgical methods

**Fig. 2:**
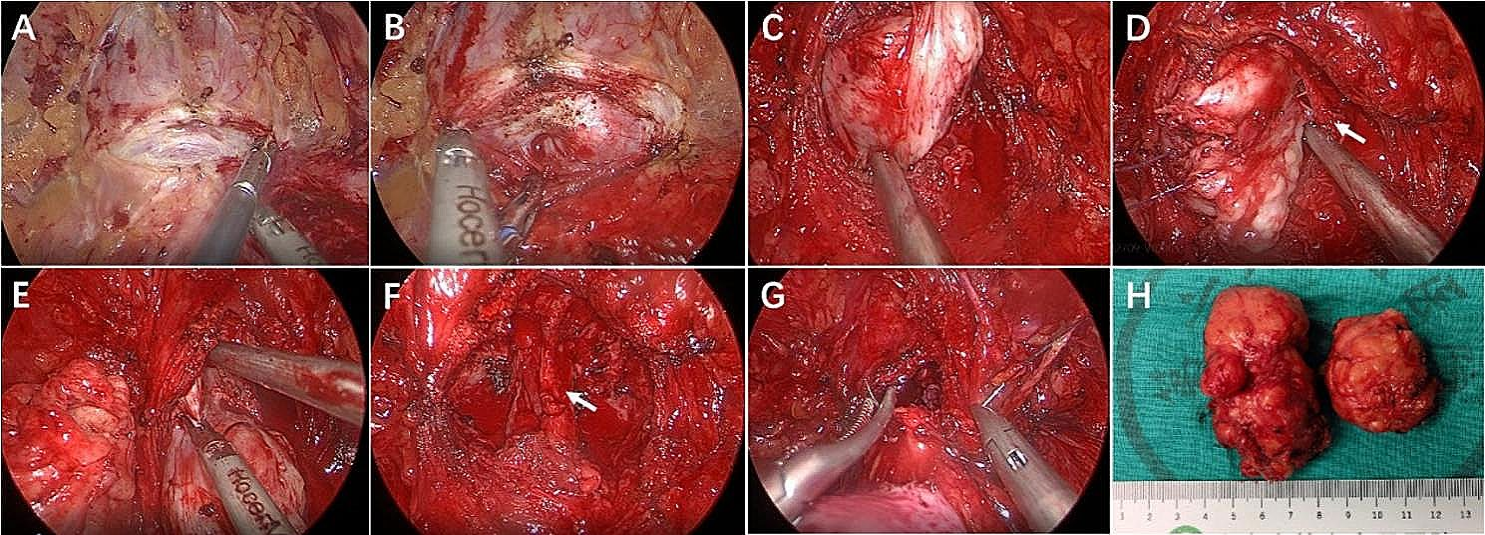
Surgical steps of US-LSP. **A**, The transversal incision near vesicoprostatic junction was performed on the anterior wall of the prostate capsule using the harmonic scalpel; **B**, Looking for the plane between the prostatic surgical capsule and hyperplastic gland; **C**, Hyperplastic gland was enucleated to reach the apex along capsule plane; **D**, Separating the hyperplastic glands from the urethra and try to preserve the integrity of the urethra (arrow denotes urethra); **E**, Dissociation of hyperplastic glands at prostatic apex; **F**, Checking the prostatic fossa to confirm that there was no residual prostate gland and hemostasis (arrow denotes urethra); **G**, The prostatic capsule was reconstructed with running 2 –0 V-lock suture; **H**, General view of enucleated glands

All procedures were performed using Laparoscopic Surgical System (Karl Storz, Tuttlingen, Germany). US-LSP has been previously described in several publications [4, 8, 10, 11]. At the beginning of the procedure, a 16Fr two-way or 18Fr three-way Foley catheter was inserted. US-LSP was performed via the extraperitoneal approach using 4 or 5 ports (three 10-mm ports and one or two 5-mm ports). A 2–3 cm longitudinal incision was made under the umbilicus for camera port placement, and a balloon dilatator was used to expose pre-peritoneal space. After removing the adipose tissue from the prostate and bladder, a transverse incision near the vesicoprostatic junction was performed on the anterior wall of the prostate capsule using the harmonic scalpel to identify the hyperplastic gland. The hyperplastic gland was enucleated using a harmonic scalpel, an aspiration cannula, and a claw grasper. This was done between the hyperplastic gland and the surgical capsule, in both the frontal and lateral planes of the prostate. Hemostasis of the surgical capsule was achieved using a harmonic scalpel or bipolar cautery. After confirming the prostate urethra near the bladder neck, the gland in front of the prostate urethra was cut into the left and right lobes. We separated the gland from the urethra of the prostate with a sharp dissection in the frontal and lateral planes of the prostate to reach the apex and tried to preserve the integrity of the urethra. Likewise, the posterior adenoma of the prostate urethra was cut in the near bladder neck. We continued to enucleate in the lateral and posterior planes of gland along the surgical capsule plane to reach the apex. Finally, the left and right lobes of gland were detached separately at the verumontanum level with a harmonic scalpel. After prostate adenoma removal, the urethral integrity is leak-tested by filling the bladder with saline. If the urethra is completely preserved or the leak is immediately repaired by a 4 –0 V-lock suture until there is no observed leakage, we decided to avoid continuous bladder irrigation. If the urethra was severely broken with significant bleeding (hematuria) and couldn’t be repaired, we decided to conversion to standard technique that does not preserve the urethra. Then, an 18Fr three-way catheter was inserted, and continuous bladder irrigation was initiated until there is no hematuria. After confirming that there was no residual hyperplastic gland and hemostasis, the prostatic capsule incision was closed by a running 2 –0 V-lock suture. When patients present with concurrent bladder stones, the stones are removed after the prostate is addressed. The bladder is filled with 200 ml of saline via the catheter and the anterior bladder wall is adequately exposed. A longitudinal opening is made on the anterior bladder wall and the stone is identified and removed. After removal of bladder stone, the incision is closed with a 2 –0 V-lock suture. The procedure was concluded with the removal of the prostate gland and bladder stones through the sub-umbilical incision, following its insertion into the specimen remover. A drainage tube was then inserted through the right 5-mm port (Fig. 2).

Upon the patient’s return to a normal diet, normal ambulation, and normal vital signs following surgery, it was determined that the patient could be discharged. The catheter is routinely removed at one week after surgery unless the degree of hematuria requires prolonged bladder catheterization.

### Follow-up

Follow-up consisted of scheduled visits at 1, 3, and 6 mo after surgery including Q_max_, IPSS, QoL, PVR, PSA, IIEF-5, and MSHQ-EjD scores. Furthermore, 16Fr flexible cystoscopy was performed at 4 wk postoperatively. For patients who underwent mp-MRI preoperatively, mp-MRI was performed again to evaluate the efficacy of prostate adenoma removal at 1 month postoperatively.

### Statistical methods

Statistical analysis was performed with SPSS 25.0 software program. Continuous variables were presented as median and interquartile range (IQR). Categorical variables are reported as frequencies (%). A nonparametric test was used with Wilcoxon signed-rank test. A probability level of *p* < 0.05 was considered significant.

## Results

Thirty-nine patients with significant BPH-related LUTS including 22 with asymptomatic anterior urethral stricture and 17 with asymptomatic posterior urethral stricture were selected and no case was excluded for the study. Baseline demographic and clinical data are summarized in Table 1. All patients were using alpha-blockers and finasteride, and 6 (15.4%) had an indwelling catheter. Twenty-six (66.7%) patients had valid sexual activity before the intervention or the date of catheterization. Among them, 25 (64.1%) patients had normal ejaculation preoperatively, including 19 cases with asymptomatic anterior urethral stricture and 6 cases with asymptomatic posterior urethral stricture. The median IIEF-5 and MSHQ-EjD score were 18 (IQR: 10–21) and 9 (IQR: 7–11) preoperatively, respectively.

**Table 1:** Baseline demographic and clinical data in US-LSP

Patient demographics (n = 39)	Results
Age (years), median (IQR)	64.5 (60–68)
BMI (kg/m²), median (IQR)	26.2 (23.1–29.5)
IIEF-5, median (IQR)	18 (10–21)
MSHQ-EjD, median (IQR)	9 (7–11)
Q_max_ (mL/s)^a^, median (IQR)	7.4 (5.5–10)
IPSS, median (IQR)	23 (20–29)
QoL, median (IQR)	5 (4–6)
PVR(mL), median (IQR)	90.4 (56–355)
PSA(ng/mL), median (IQR)	5.1 (3.1–10.3)
TRUS(mL), median (IQR)	104.5 (81–129)
Median lobes, n (%)	6(15.4)
Median lobe length (cm), median (IQR)	1.1(0.7–1.5)
BPH-related complications, n (%)	
Urinary retention/indwelling catheter	6 (15)
Bladder stone	3 (8)
Recurrent urinary tract infection	11(28)
Recurrent hematuria	5 (13)
Hydronephrosis	1 (3)
Bladder diverticulum	1 (3)

BMI, Body Mass Index; Qmax, maximun flow rate; IPSS, International Prostate Symptom Score; QoL, quality of life; PVR, post-voided residual volume; PSA, prostate-specific antigen; TRUS, Transrectal prostate ultrasound (prostate volume); IIEF-5, International Index of Erectile Function-5; MSHQ-EjD, Male Sexual Health Questionnaire to assess ejaculatory dysfunction

^a^Excludes 6 patients with Foley catheter or otherwise unable to void

Perioperative characteristics and postoperative complications according to the Clavien-Dindo classification are reported in Table 2. Median operative time was 118 min (IQR: 100–145). Median estimated blood loss was 224 ml (IQR: 190–255). Continuous bladder irrigation was avoided in 33 patients (84.6%). Postoperative complications occurred in 5 patients (12.8%), including 4 cases with Clavien-Dindo grade 1 (2 cases with transient hematuria and 1 case with wound infection) and grade 2 (1 case requiring blood transfusion) and 1 case with Clavien-Dindo grade 3a (requiring endoscopy reintervention because of gross hematuria).

**Table 2:** Perioperative characteristics and postoperative complications according to Clavien-Dindo classification

Parameter	Results
Patients(n)	39
Surgery time (minutes), median (IQR)	118 (100–145)
Prostate enucleated weight (grams), median (IQR)	68.2 (52–84)
Estimated blood loss (ml), median (IQR)	224 (190–255)
Continuous bladder irrigation, n (%)	6(15.4)
Hospital Stay (days), median (IQR)	4 (3–5)
Catheterization time (days), median (IQR)	7 (6–8)
Conversion to standard technique, n (%)	3 (7.7)
Symptomatic urethral stricture need treatment, n (%)	0
Incidental prostate cancer, n (%)	0
SUI, n (%)	2/39(5.1%)
Retrograde ejaculation, n (%)	3/25(12%)
Postoperative complications, n (%)	
Grade 1	3 (7.6%)
Grade 2	1 (2.6%)
Grade 3a	1 (2.6%)
Grade 3b	0
Grade 4	0

SUI, stress urinary incontinence. Standard technique refers to laparoscopic simple prostatectomy that does not preserve the urethra

**Fig. 3:**
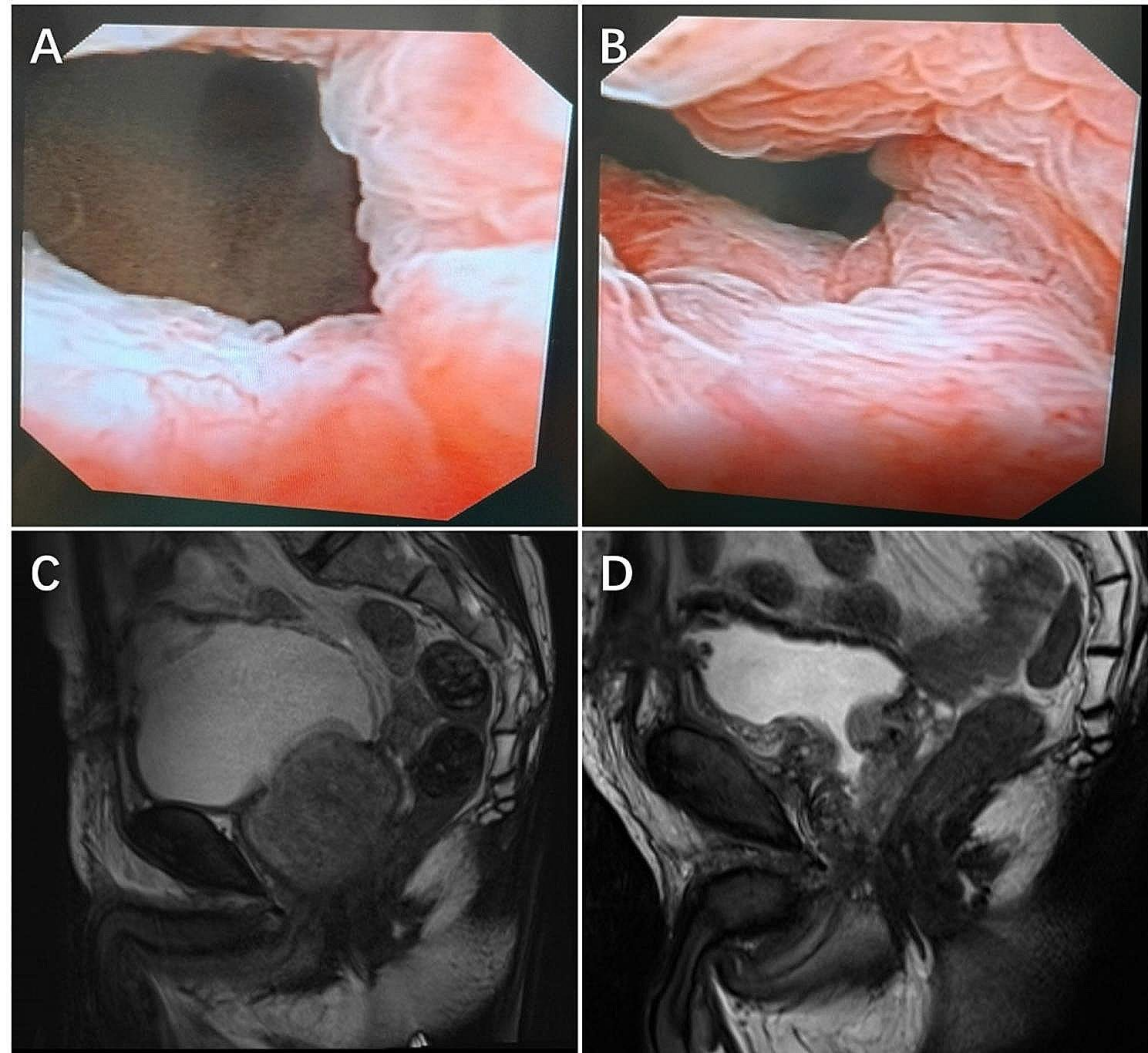
**A** and **B**, Postoperative flexible cystoscopy revealed that bladder neck and urethra were unobstructed, and the mucosa at bladder neck and prostatic urethra was intact; **C**, Preoperative mp-MRI showed a large and obstructive prostatic adenoma; **D**, postoperative mp-MRI revealed that the grands were completely removed and urethras were unobstructed and intact

At 1 month follow-up, 3 (12%) patients complained of retrograde ejaculation and 2 (5.1%) patients reported stress urinary incontinence (SUI). Postoperative flexible cystoscopy revealed the integrity of the mucosa at the bladder neck and prostatic urethra without extrinsic compression deforming its walls (Fig. 3A **and B**). Eight (20.5%) patients underwent prostate mp-MRI due to elevated PSA, and mp-MRI showed a large and obstructive prostatic adenoma (Fig. 3C). Among them, six patients underwent prostate mp-MRI at 1 month postoperatively, revealing that the adenomas were completely removed and the urethras were unobstructed and intact (Fig. 3D). Three patients underwent prostate biopsy due to elevated PSA preoperatively. US-LSP was performed at 1 month after the prostate biopsy and biopsy did not affect the plane of enucleation and rate of complications. At 6 months, there was no case of SUI. No patient reported an aggravated urethral stricture needing repeat treatment during follow-up. At each time point during follow-up, US-LSP presented significant improvements when compared to the baseline in terms of IPSS, QoL, Qmax, and PVR (*P* < 0.05, Table 3). However, no differences were found between pre- and postoperative values of IIEF-5 and MSHQ-EjD (*P*>0.05, Table 3). No prostate cancer was reported by pathological diagnosis.

**Table 3:** Functional outcomes after US-LSP

	Baseline	1 mo	3 mo	6 mo	Baseline vs. 1 mo	Baseline vs. 3 mo	Baseline vs. 6 mo
Q_max_(mL/s), median (IQR)	7.4 (5.5–10)	23.6(21-26.5)	25.1(22.3–27.1)	25.8(23-27.9)	< 0.0001	< 0.0001	< 0.0001
IPSS, median (IQR)	23 (20–29)	7(5–11)	6 (5–10)	5(4–10)	< 0.0001	< 0.0001	< 0.0001
QoL, median (IQR)	5 (4–6)	2 (1–2)	1 (0–1)	1 (0–2)	< 0.0001	< 0.0001	< 0.0001
PVR(mL), median (IQR)	90.4 (56–355)	11.2(5–20)	12.3(7–18)	10.6(6–21)	< 0.0001	< 0.0001	< 0.0001
PSA(ng/mL), median (IQR)	5.1 (3.1–10.3)	1.5(0.8–2.4)	1.4(0.9–2.8)	1.2(0.6–2.2)	< 0.0001	< 0.0001	< 0.0001
IIEF-5, median (IQR)	18 (10–21)	17 (10–22)	18 (11–21)	17 (13–21)	0.363	0.659	0.316
MSHQ-EjD, median (IQR)	9 (7–11)	10 (5–12)	11 (7–13)	11 (6–15)	0.108	0.073	0.085

Q_max_, maximun flow rate; IPSS, International Prostate Symptom Score; QoL, quality of life; PVR, post-voided residual volume; PSA, prostate-specific antigen; IIEF-5, International Index of Erectile Function-5; MSHQ-EjD, Male Sexual Health Questionnaire to assess ejaculatory dysfunction

## Discussion

This study is the first to evaluate the effects of US-LSP in the treatment of large-volume BPH with asymptomatic urethral stricture after urethral stricture surgery. According to our research results, US-LSP was a safe and effective procedure that significantly improves LUTS in large-volume BPH. Meanwhile, US-LSP could reduce the risk of SUI in patients with a history of posterior urethral surgery. These results were in line with previous studies [4, 9, 13].

Surgical methods for BPH mainly include transurethral resection of the prostate (TURP), transurethral laser enucleation of the prostate, transurethral laser vaporization of the prostate, OP, and minimal invasive simple prostatectomy (MISP). Prostate volume is a critical factor influencing the choice of surgical methods [6]. According to updated EAU guidelines on the management of non-neurogenic male LUTS, OP and endoscopic enucleation of the prostate (EEP) remain standard treatments for patients with larger prostatic glands [6]. Because simple prostatectomy avoids the transurethral procedure of EEP, the technique is particularly suitable for large-volume BPH patients with concomitant asymptomatic urethral stricture. Compared to OP, MISP including LSP and RASP has the advantage of decreased perioperative complications and as a minimally invasive treatment method has been unanimously recognized for its safety and effectiveness in the treatment of large-volume BPH [14–16]. Therefore, in this study, for large-volume BPH with asymptomatic urethral stricture, MISP was employed as the treatment method.

Despite recent technical innovations of MISP, several postoperative problems are still unresolved. Among them, retrograde ejaculation is one of the most frequent problems, which has a major impact on the quality of life in BPH patients, especially in young and sexually active men [17, 18]. To decrease postoperative retrograde ejaculation rates, Quan et al. described, for the first time, US-LSP, which involves the preservation of the prostate urethra and bladder neck, to maintain anterograde ejaculation in the treatment of large-volume BPH following urethral-sparing OP, as described by Dixon et al. in 1990 [10, 19].

In current study, US-LSP could maintain ejaculatory function in a high percentage of patients. The exact mechanism of postoperative retrograde ejaculation remains unclear. It has been suggested that retrograde ejaculation may be caused by the impaired closure mechanism of the bladder neck [20]. Indeed, complete preservation of the bladder neck, together with the parafollicular area, is critical and advocated when attempting an ejaculation function-sparing technique endoscopically [20, 21]. Porpiglia et al. found that the absence of urethral infraction was a predictor of ejaculation recovery, which can be explained by the fact that the urethra underwent a remodeling process that can potentially cause edema or alter the physiological contractions of the urethra during ejaculation in the first months postoperatively [16]. Thus, the low rate of retrograde ejaculation (12%) observed in the current study can be attributed to US-LSP, which preserves the urethra and bladder neck.

Preservation of the prostate urethra not only can improve the rate of antegrade ejaculation postoperatively, but also minimize surgical trauma and perioperative complications [20]. Meanwhile, urethral preservation can reduce the rate of postoperative bladder irrigation, leading to shorter catheterization time, and possibly decreasing the risk of symptomatic urethral stricture [14, 20]. In current study, continuous bladder irrigation was avoided in 33 (84.6%) patients, and no patient reported aggravated urethral stricture and postoperative complications occurred in only 5 (12.8%) patients, including 4 cases with Clavien-Dindo grade 1 and grade 2 and 1 case with grade 3a, which was attributed to the urethral preservation in the US-LSP. Meanwhile, the incidence of postoperative complications was lower than that reported in previous literature on LSP and EEP [22–24].

We should realize that BPH patients with a history of posterior urethroplasty have an altered anatomy and urinary continence is tenuous because the external sphincter function was usually compromised by the urethroplasty [1]. The primary continence is maintained by the internal sphincter located at the bladder neck. Therefore, traditional endoscopic surgery carries a high risk of urinary incontinence because of the destruction of the internal sphincter mechanism in BPH patients with a history of posterior urethroplasty [25, 26]. For this reason, experts recommend treating these patients with medical therapy unless BPH surgical treatment is absolutely indicated [27]. Therefore, the patients of the current study required surgical treatment after patients failed medical management of their LUTS. In our study, at 1-month follow-up, only one (5.9%) patient complained of SUI in 17 patients with asymptomatic posterior urethral stricture after posterior urethral stricture surgery, and there was no case of SUI at 6 months, which confirms that preservation of bladder neck is a critical factor for maintaining the internal sphincter mechanism.

In the current study, 16Fr flexible cystoscopy evaluation confirmed that calibre of the urethral lumen was greater than 16Fr, and the diagnosis of BPH was established after excluding neurogenic dysfunction according to EAU guidelines on urethral strictures [2]. Thus, patients with asymptomatic urethral stricture after urethral stricture surgery did not require repeat urethral surgery but needed BPH surgical treatment because of significant BPH-related LUTS. Our results showed that US-LSP presented significant improvements when compared to baseline in terms of IPSS, QoL, Q_max,_ and PVR during follow-up, which further confirmed that significant LUTS of patients should be contributed to BPH, not asymptomatic urethral stricture.

Our study has some limitations. Firstly, the limited number of patients is major limitation. Secondly, the relatively short follow-up period for BPH patients with asymptomatic urethral stricture is also a limitation. At last, all surgeries in this study were performed by highly experienced laparoscopic surgeons, so the results cannot be generalized to all clinical centers.

## Conclusion

Our study confirmed that US-LSP has achieved good results in improving LUTS of large-volume BPH with asymptomatic urethral stricture after urethral stricture surgery. Additionally, US-LSP could reduce the risk of SUI in patients with a history of posterior urethral stricture surgery and maintain ejaculatory function in a high percentage of patients. US-LSP should be considered as an option for large-volume BPH patients, especially in young and sexually active men with asymptomatic urethral stricture after posterior urethral stricture surgery.

## Data Availability

The data sets used and/or analyzed during the current study are available from the corresponding author on reasonable request.

## Abbreviations

US-LSP: urethral-sparing laparoscopic simple prostatectomy
BPH: benign prostatic hyperplasia
LUTS: lower urinary tract symptoms
IQR: interquartile range
SUI: stress urinary incontinence
EAU: European Association of Urology
HoLEP: holmium laser enucleation of prostate
OP: open simple prostatectomy
LSP: laparoscopic simple prostatectomy
RASP: robotic-assisted simple prostatectomy
EjD: ejaculatory dysfunction
BMI: body mass index
Q_max_: maximum urinary flow rate
IPSS: International Prostate Symptom Score
QoL: Quality of life
PVR: postvoid residual urine
PSA: prostate specific antigen
IIEF-5: International Index of Erectile Function-5 questionnaire
TURP: transurethral resection of the prostate
MISP: minimal invasive simple prostatectomy
EEP: endoscopic enucleation of the prostate

## References

[1] Mishra K, Baeza C, Bukavina L, Gómez RG (2019). Modified transurethral resection of the prostate for the management of BPH-related refractory lower urinary tract symptoms in patients with a history of pelvic fracture urethral injury reconstruction. Int Urol Nephrol.

[2] Lumen N, Campos-Juanatey F, Dimitropoulos K, Greenwell T, Martins FE, Osman N et al. EAU Guidelines on Urethral Strictures. 2023. https://uroweb.org/guidelines/urethral-strictures.

[3] Kim JH, Park JY, Shim JS, Lee JG, Moon du G, Yoo JW (2014). Comparison of outpatient versus inpatient transurethral prostate resection for benign prostatic hyperplasia: comparative, prospective bi-centre study. Can Urol Assoc J.

[4] Gunseren KO, Akdemir S, Çiçek MC, Yıldız A, Arslan M, Yavaşcaoğlu İ (2021). Holmium Laser Enucleation, laparoscopic simple prostatectomy, or open prostatectomy: the role of the prostate volume in terms of Operation Time. Urol Int.

[5] Zell MA, Abdul-Muhsin H, Navaratnam A, Cumsky J, Girardo M, Cornella J (2021). Holmium laser enucleation of the prostate for very large benign prostatic hyperplasia (≥ 200 cc). World J Urol.

[6] Gravas S, Cornu JN, Gacci M, Gratzke C, Herrmann TRW, Mamoulakis C et al. EAU guidelines on Management of Non-Neurogenic Male Lower Urinary Tract Symptoms (LUTS), incl. Benign Prostatic Obstruction (BPO). 2023. http://uroweb.org/guideline/treatment-of-non-neurogenic-male-luts/.

[7] Pariser JJ, Pearce SM, Patel SG, Bales GT (2015). National Trends of Simple Prostatectomy for Benign Prostatic Hyperplasia with an analysis of risk factors for adverse perioperative outcomes. Urology.

[8] Lombardo R, Zarraonandia Andraca A, Plaza Alonso C, González-Dacal JA, Rodríguez Núñez H, Barreiro Mallo A (2021). Laparoscopic simple prostatectomy vs bipolar plasma enucleation of the prostate in large benign prostatic hyperplasia: a two-center 3-year comparison. World J Urol.

[9] Uschi A, Al Salhi Y, Velotti G, Capone L, Martoccia A, Suraci PP (2021). Holmium laser enucleation of prostate versus minimally invasive simple prostatectomy for large volume (≥ 120 ml) prostate glands: a prospective multicenter randomized study. Minerva Urol Nefrol.

[10] Quan C, Chang W, Chen J, Li B, Niu Y (2011). Laparoscopic Madigan prostatectomy. J Endourol.

[11] Wang P, Xia D, Ye S, Kong D, Qin J, Jing T (2018). Robotic-assisted urethra-sparing simple Prostatectomy Via an Extraperitoneal Approach. Urology.

[12] Kowalewski KF, Hartung FO, von Hardenberg J, Haney CM, Kriegmair MC, Nuhn P (2022). Robot-assisted simple prostatectomy *vs* endoscopic enucleation of the prostate: a systematic review and Meta-analysis of comparative trials. J Endourol.

[13] Abi Chebel J, Sarkis J, El Helou E, Hanna E, Abi Tayeh G, Semaan A (2020). Minimally invasive simple prostatectomy in the era of laser enucleation for high-volume prostates: a systematic review and meta-analysis. Arab J Urol.

[14] Cardoso A, Lima E (2021). Urethra-sparing minimally invasive simple prostatectomy: an old technique revisited. Curr Opin Urol.

[15] Kiraç M, Ergin G, Kibar Y, Köprü B, Biri H (2021). Robotic simple prostatectomy is a safe and effective technique for benign prostatic hyperplasia: our single center initial short-term follow-up results for 42 patients. Turk J Urol.

[16] Porpiglia F, Checcucci E, Amparore D, Niculescu G, Volpi G, Piramide F (2021). Urethral-sparing Robot-assisted simple prostatectomy: an innovative technique to preserve ejaculatory function overcoming the limitation of the Standard Millin Approach. Eur Urol.

[17] Rowland D, McMahon CG, Abdo C, Chen J, Jannini E, Waldinger MD (2010). Disorders of orgasm and ejaculation in men. J Sex Med.

[18] Marra G, Sturch P, Oderda M, Tabatabaei S, Muir G, Gontero P (2016). Systematic review of lower urinary tract symptoms/benign prostatic hyperplasia surgical treatments on men’s ejaculatory function: time for a bespoke approach?. Int J Urol.

[19] Dixon AR, Lord PH, Madigan MR (1990). The Madigan prostatectomy. J Urol.

[20] Simone G, Misuraca L, Anceschi U, Minisola F, Ferriero M, Guaglianone S, Tuderti G, Gallucci M (2019). Urethra and ejaculation preserving Robot-assisted simple prostatectomy: Near-infrared fluorescence imaging-guided Madigan Technique. Eur Urol.

[21] Sturch P, Woo HH, McNicholas T, Muir G (2015). Ejaculatory dysfunction after treatment for lower urinary tract symptoms: retrograde ejaculation or retrograde thinking?. BJU Int.

[22] Liu S, Zhou L, Wang J, Tan Y, Huang T, Xiao J (2022). Extraperitoneal laparoscopic simple prostatectomy with urethra preservation using urethral initiation as the entry point: a practical approach for the treatment of benign prostatic obstruction. World J Urol.

[23] Zarraonandia Andraca A, Lombardo R, Carrion Valencia A, González-Dacal JA, Rodríguez Núñez H, Samper Mateo P, Sica A, Tema G, DE Nunzio C, Tubaro A, Ruibal Moldes M (2021). Laparoscopic simple prostatectomy: a large single-center prospective cohort study. Minerva Urol Nephrol.

[24] Habib E, Abdallah MF, ElSheemy MS, Badawy MH, Nour HH, Kamal AM, AbdelMohsen M, Roshdy MA, Meshref A (2022). Holmium laser enucleation versus bipolar resection in the management of large-volume benign prostatic hyperplasia: a randomized controlled trial. Int J Urol.

[25] Koraitim MM, Atta MA, Fattah GA, Ismail HR (2003). Mechanism of continence after repair of post-traumatic posterior urethral strictures. Urology.

[26] Whitson JM, McAninch JW, Tanagho EA, Metro MJ, Rahman NU (2008). Mechanism of continence after repair of posterior urethral disruption: evidence of rhabdosphincter activity. J Urol.

[27] Gomez RG, Scarberry K (2018). Anatomy and techniques in posterior urethroplasty. Transl Androl Urol.

